# Ethnomedicinal Uses of Thai Legumes (*Fabaceae*) and Their Potential in Industrial Applications

**DOI:** 10.1002/fsn3.72343

**Published:** 2026-09-27

**Authors:** Muhammad Tayyab Arshad, Farhang Hameed Awlqadr, Iffat Ullah, Mamoona Shehzadi, Aneeq Ahmad, Ali Ikram, Maneeba Khalid, Md. Sakhawot Hossain

**Affiliations:** ^1^ Functional Food and Nutrition Program, Faculty of Agro‐Industry Prince of Songkla University Hat Yai, Songkhla Thailand; ^2^ University Institute of Food Science and Technology The University of Lahore Lahore Pakistan; ^3^ Food Science and Quality Control, Halabja Technical College Sulaimani Polytechnic University Sulaymaniyah Iraq; ^4^ Department of Pharmaceutical Chemistry, Faculty of Pharmaceutical Sciences Prince of Songkla University Hat Yai, Songkhla Thailand; ^5^ Drug Delivery System Excellence Center, Faculty of Pharmaceutical Sciences Prince of Songkla University Hat Yai, Songkhla Thailand; ^6^ Department of Food Science Government College University Faisalabad Faisalabad Pakistan; ^7^ College of Pharmaceutical Science Zhejiang University of Technology Hangzhou People's Republic of China; ^8^ University Institute of Dietetics and Nutrition Sciences The University of Chenab Gujrat Pakistan; ^9^ Department of Nutrition and Food Engineering Daffodil International University Dhaka Bangladesh

**Keywords:** ethnomedicinal, nitrogen fixation, pharmacology, phytochemicals, Thai legumes

## Abstract

The *Fabaceae* legumes form part of the global food systems, traditional medicines and provide a good source of proteins, dietary fiber, vitamins, minerals and a wide range of bioactive compounds. *Glycine max, Vigna radiata, Cajanus cajan, Clitoria ternatea* and *Vigna unguiculata* are examples of legumes that are important in nutrition, ethnomedicine and sustainable agriculture in Thailand. This review gives a general overview of the phytochemical composition, pharmaceutical benefits and industrial use of Thai legumes. These species contain phenolics, flavonoids, isoflavones, saponins and bioactive peptides which form the basis of their antioxidant, anti‐inflammatory, antidiabetic, antimicrobial, cardioprotective, anticancer and neuroprotective effects. In Thailand, traditional knowledge systems emphasize that they are used in the treatment of various ailments and therefore they are pharmacologically relevant. Thai legumes show great promise in the industrial environment, in addition to their medicinal value, in a variety of industries. They are used as primary ingredients in functional foods, plant‐based proteins and fermented foods in the food industry and have the advantage of using high processing technologies, including extrusion and solid‐state fermentation. In pharmaceuticals, their bioactive compounds are developed in nutraceutical and drugs development. Also, their use in cosmetic formulations and biodegradable biomaterials is an indication of increased interest in sustainable and natural products. Legumes also play a vital role in agriculture by fixing biological nitrogen in the soil making the soil fertile and environmentally friendly. These benefits notwithstanding, several challenges continue to constrain the field, including a shortage of clinical validation, marked phytochemical variability and limited scalability of processing. The review indicates that there is a need to combine research methods in order to harness all the medicinal and industrial potential of Thai legumes.

## Graphical abstract

Thai legumes (Fabaceae) are rich sources of bioactive phytochemicals, including saponins, flavonoids, biochanin A, and daidzein. These compounds contribute to diverse ethnomedicinal properties, including antioxidant, antiviral, anti‐inflammatory, anticancer, and cardioprotective activities. Their nutritional and functional potential further supports applications in the food, animal feed, pharmaceutical, and cosmetic industries, highlighting their promising value for sustainable utilization.

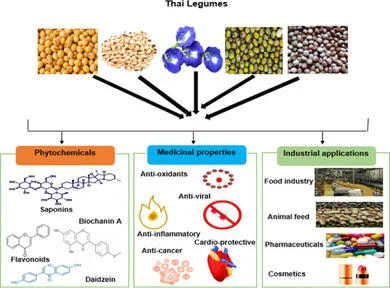

## 1 Introduction

Legumes, members of the family Fabaceae, are recognized worldwide as fundamental components of both the human diet and traditional medicine. Their protein content, which typically ranges from 20% to 35%, is complemented by carbohydrates, dietary fiber, vitamins and minerals, contributing to their cultivation across diverse regions of the world. This nutritional composition helps substantiate the importance of these foods as essential vegetarian products, which are useful in the regulation of blood sugar, cardiovascular health and weight. Moreover, legumes are also enriched with numerous bioactive and non‐nutritive compounds, such as antioxidants, bioactive peptides, among others, that are related to their health promotional effects, including antidiabetic, anti‐inflammatory and anti‐hypertensive effects. It has been demonstrated that such compounds are modified to enhance the nutritional quality of traditional methods of processing and such practices should be considered important in homes where legumes are the staple food (Didinger and Thompson 2021; Grdeń and Jakubczyk 2023; Gulewicz et al. 2014).

Besides dietary uses, Fabaceae species have had an extensive ethnopharmacological history that has been followed with various medicinal applications all over the world. As seen by ethnobotanical research (e.g., Zimbabwe), as well as in other parts of the world, numerous species of Fabaceae are used to treat many ailments, including gastrointestinal, respiratory, reproductive, and sexually transmitted diseases, traditionally such as Indigofera, Senna, and Albizia. These applications entail various parts of the plant such as roots, leaves, bark, and fruits and are backed by evidence in the pharmacological and phytochemical studies that reinforce their therapeutic benefits. Nonetheless, a lack of toxicological evaluation and mechanistic investigation suggests the need to conduct additional systematic studies that will scientifically prove these customary practices (Maroyi 2023).

Thailand, being a part of Southeast Asia, where plants like *Pongamia pinnata* can be found, may represent an interesting setting of these legumes due to their diverse uses outside of nutrition and medicine. An example of medicinal, traditional and industrial usage is *Pongamia pinnata* : an important source of bioactive compounds in Ayurvedic medicine and prized as a source of biofuel because of its rich composition of seeds in terms of oil. The species is also used to restore land and local economies, which is an experience of the socio‐economic and environmental benefits of Thai legumes. However, the variety of the use of such species in Thailand stays uninvestigated, particularly in the context of combined discovery of medicinal activity and new industrial applications (Vidhale et al. 2024).

Although there have been numerous studies on the legumes (Fabaceae) around the world, there is an apparent lack in the integrated review that effectively covers both medicinal and industrial applications especially in the Thai context. There is also inaccurate terminology and category of legumes, which makes it hard to harmonize dietary guidelines and alleviate health messages to people. In addition, genetic resources stored in secondary and tertiary gene pools are underutilized to enhance variety development and in bioprospecting, which further highlights the need to explore the areas (Didinger and Thompson 2021; Singh et al. 2007).

Based on this, the purpose of a dedicated study of Thai legumes within the family Fabaceae should be regarded as the systematic listing of phytochemical and pharmacological information, a critical assessment of safety and toxicological characteristics and an analysis of their application in industry, that is, biofuels and nutraceuticals. The review would be intended to elucidate and standardize terminology to reflect world dietary frameworks, support genetic diversity evaluation to improve crops and indicate the recent technological progress, for example, Phyto some‐delivery system, which leads to the bioavailability of compounds in legumes. Close to the traditional knowledge, such a review would fill in the existing gaps and explain the full potential of the Thai legumes in terms of nutrition, medicine and sustainable industry (Singh et al. 2007; Yadav et al. 2025).

To conclude, the Fabaceae family of legumes plays vital functions both globally and in Thailand in the nutritional, traditional and industrial sectors. Their bioactive profiles are rich and have great health benefits; however, it needs more scientific validation and merging of ethnomedical with the emerging industrial practices. Thai legumes have not received the level of attention they deserve, and hence, an integrated review would be helpful in creating solutions that would lead to their maximum exploitation in terms of health, economic sustainability and ecological stability.

### 1.1 Methodology and Search Strategy

This narrative review was compiled through a structured search of the peer‐reviewed literature published between 2000 and 2025. The Scopus, Web of Science, PubMed, and Google Scholar databases were queried using combinations of the keywords “Thai legumes”, “Fabaceae”, “ethnomedicine”, “phytochemicals”, “nitrogen fixation”, and “functional foods”, together with the individual species names ( *Glycine max* , *Vigna radiata* , *Cajanus cajan* , *Clitoria ternatea* , and *Vigna unguiculata* ). Records were included when they reported ethnomedicinal, phytochemical, pharmacological, agricultural, or industrial data relevant to legumes cultivated or traditionally used in Thailand and neighboring Southeast Asian regions. Non‐English records, conference abstracts lacking primary data, and studies unrelated to the Fabaceae were excluded. Where Thailand‐specific experimental data were unavailable, findings from taxonomically related species were used with caution and are explicitly identified as such throughout the text.

## 2 Distribution of Thai Legumes

Thai legumes are known to be extremely diverse and distributed across a broad taxonomic and ecological range and have a major presence of *Glycine max* (soybean), *Vigna radiata* (mung bean), *Cajanus cajan* (pigeon pea), *Clitoria ternatea* (butterfly pea) and *Vigna unguiculata* (cowpea). Taxonomically and botanically, legumes in Thailand are incorporated in a large and complicated family of Fabaceae, with several subfamilies, including Caesalapinioideae, Mimosoideae and Papilionoideae. Recent phylogenomic studies have narrowed down the groups and there have been new higher‐level taxonomies which are better at helping identify and communicate the groups to the researchers and practitioners. These updated categories consider the high morphological and ecological diversity within legumes, such as woody, shrub and herbaceous plants that are typical of the flora of Thailand (Bruneau et al. 2024).

Attending to genus Vigna, Thais are known to be a center of diversity especially to section Angulares of Asian Vigna species inclusive of *V. radiata* , *V. unguiculata* and other species. Genetic studies have been conducted using AFLP that has found out that there is a considerable amount of genetic variation in the wild and cultivated populations in Thailand and other nearby areas. As an example, Vigna umbellate (rice bean), seems to have experienced one domestication event, probably in the north of Thailand and neighboring Myanmar. Northern Thais *Vigna mungo* (black gram) has genetically separate wild populations that are not related to Indian and other cultivated mungo populations in Thailand and this highlights the existence of unique germplasm which may be of genetically useful breeding and conservation value. On the other hand, genetic correlation of certain species, that is, *V. umbellate*, shows that they have not differentiated much with their wild and weedy counterparts in Thailand (Seehalak et al. 2006).

There are the major legume species of Thais with various applications and important cultural values. *Glycine max* (soybean) is a very important crop in the country of northern Thailand, especially to the Karen people, who use it both as food and as medicine. Such ethnobotanical role is consistent with the general trends in which legumes are used as staples, medicine, and raw material in the local society. The data document of the Karen community 83 legume species in 45 genera have various usages in nutrition, traditional medicine, and material culture; this brings into focus the versatility and cultural embeddedness of the legumes in Thailand (Sutjaritjai et al. 2019). Ecologically, the Thai legumes are also very common in different habitats such as the pine‐wiregrass ecosystems that are commonly burnt and, ecologically, legumes play a very significant role in the ecological processes, including fixation of nitrogen and nutrient cycling. Abiotic factors like moisture content of soil, availability of light, and mineral content of soil affect the abundance of species and distribution patterns. The highest abundance of legumes occurs in rainy season (June), which demonstrates their ability to adapt to seasonal climatic change that is common in Thailand (Hainds et al. 1999).

Symbiosis with nitrogen‐fixing bacteria also determines the diversity of legumes and the ecological success. The Mesorhizobium species are the major modulators of native legumes in Thai ecosystems and they form root nodules where biological fixation of nitrogen takes place. On the other hand, the introduced legume species tend to interact with Bradyrhizobium species. Such symbiotic specificity also influences adaptability and distribution of legume species and gives indication of coevolutionary dynamic topography between legumes and symbiont bacteria in Thailand (Weir et al. 2004).

Overall, the genera, species, and ecology of Thai legumes are very diverse and include many important species, including *Glycine max* , *Vigna radiata* , *Cajanus cajan* , *Clitoria ternatea* , and *Vigna unguiculata* , which have an important contribution to nutrition, traditional medicine, and ecosystem functions. These two facts indicate Thailand as a hub of legume biodiversity due to the high genetic variability of Vigna species and their distinct histories of domestication. Moreover, the combination of ethnobotanical experience of the indigenous people and the understanding of symbiotic relationships with nitrogen‐fixing bacteria gives a complete picture of the biological and cultural relevance of Thai legumes. It is a comprehensive approach to conservation, breeding, and sustainable use of the unique legume flora of Thailand (Bruneau et al. 2024; Hainds et al. 1999; Seehalak et al. 2006; Sutjaritjai et al. 2019; Weir et al. 2004).

### 2.1 Ethnobotanical Surveys in Thailand

Dedicated ethnobotanical surveys reinforce the cultural centrality of legumes across different regions of Thailand. In the north, Sutjaritjai et al. (2019) documented 83 leguminous species belonging to 45 genera among the Karen communities, used variously as food, traditional medicine and raw materials, underscoring the depth of indigenous knowledge attached to the family. At the national scale, a systematic review of medicinal legumes by Sutjaritjai et al. (2022) compiled 261 species from 98 genera drawn from 152 sources, applying quantitative indices (informant consensus factor, use value and fidelity level) and identifying high‐use species such as *Biancaea sappan*, *Cassia fistula* and *Senna siamea* as strong candidates for public herbal‐medicine development. In the northeast (Isan), Saensouk et al. (2025) recorded 83 Fabaceae taxa across 52 genera in Maha Sarakham Province, where *Vigna unguiculata* subsp. *sesquipedalis*, *Arachis hypogaea* , *Glycine max* and *Sesbania grandiflora* showed the highest species‐use and citation values, while *Abrus precatorius* and *Albizia lebbeck* recorded maximal fidelity levels for skin ailments. Consistent with these surveys, soybean ( *Glycine max* ) holds a dual dietary and medicinal role, butterfly pea ( *Clitoria ternatea* ) is widely used as a natural colorant and traditional remedy, and *Cajanus cajan* and *Vigna* species feature in local practice for gastrointestinal and inflammatory complaints. Although quantitative ethnobotanical indices are increasingly being applied in individual provinces, systematic documentation across Thailand's diverse regions and ethnic groups remains incomplete. Expanding and standardizing region‐specific surveys would consolidate this fragmented knowledge and help prioritize species for pharmacological validation. Representative Thailand‐focused studies are summarized in Table 1.

**TABLE 1:** Representative Thailand‐focused studies on legumes (*Fabaceae*): Legume species examined, key methods and main findings.

Study focus & region	Thai legume species studied	Key methods	Main findings/achievements	References
Ethnobotanical survey among Karen communities, northern Thailand	83 leguminous species across 45 genera, including *Glycine max* , *Clitoria ternatea* , *Cajanus cajan* and *Vigna* spp	Field ethnobotanical documentation and community interviews	Extensive food, medicinal and material uses recorded; soybean holds a dual dietary‐medicinal role; butterfly pea used as a natural colorant and traditional remedy	Sutjaritjai et al. (2019)
Nationwide review of medicinal legumes, Thailand	261 species from 98 genera (e.g., *Biancaea sappan*, *Cassia fistula* , *Senna siamea* )	Systematic compilation of 152 literature sources (1986–2019) with quantitative indices (informant consensus factor, use value, fidelity level)	5387 use reports covering 420 disorders; 15 high‐use‐value species identified as priority candidates for public herbal‐medicine development	Sutjaritjai et al. (2022)
Ethnobotanical survey of Fabaceae, Maha Sarakham Province (northeastern/Isan Thailand)	83 taxa across 52 genera, including *Vigna unguiculata* subsp. *sesquipedalis*, *Arachis hypogaea* , *Glycine max* , *Sesbania grandiflora* ; medicinal *Abrus precatorius* , *Albizia lebbeck*	Field surveys and semi‐structured interviews with 260 informants; SUV, RFC, fidelity level and informant consensus factor	Highest use and citation values for cultivated food legumes; strong medicinal consensus for skin ailments; 44 taxa newly recorded for the province	(Saensouk et al. 2025)
Genetic diversity of Vigna germplasm, Thailand and neighboring regions	*Vigna radiata* , *V. unguiculata* , *V. mungo* , *V. umbellata*	AFLP marker analysis of wild and cultivated populations	High genetic variation and unique germplasm; a single domestication event for rice bean in northern Thailand/Myanmar; notable conservation and breeding value	(Seehalak et al. 2006)
On‐farm legume adoption in maize‐based systems, northern Thailand	Rice bean ( *Vigna umbellata* )	Farm‐level assessment of adoption constraints and potentials	Legume integration improves soil health and offers a viable rotational alternative to maize monoculture; adoption limited by market price and labour factors	(Yap et al. 2016)

## 3 Cultivation and Economic Importance of Thai Legumes in Thailand

The importance of legume farming in Thai agriculture is that the legumes contribute to food security, soil health, and livelihood of the rural populations. Some of the legumes that are significant in cropping systems include soybean ( *Glycine max* ), mung bean ( *Vigna radiata* ), pigeon pea ( *Cajanus cajan* ), butterfly pea ( *Clitoria ternatea* ), and cowpea ( *Vigna unguiculata* ), that are known to be rich in protein and nutritional value. They increase the soil fertility by biological fixing of nitrogen (BNF) so that they do not rely on synthetic nitrogen fertilizers and can practice sustainable agricultural practices (Gogoi et al. 2018; Rajput et al. 2024).

The agricultural activities have had to adapt to the nature of the Thai climate which is prone to flooding, hot weather and poor soil characteristics. The farmers incorporate legumes in crop rotation, rely on crop and intercropping systems to maximize land utilization, enhance soil organic matter and control pests and diseases in environmentally friendly manners. As an example, a monoculture of maize can be provided with profitability by reducing the costs of inputs and implementing monoculture, where the introduction of a legume like rice bean (Vigna umbellate) can reduce some of the yields and provide farmers with a viable alternative (Yap et al. 2016). In addition, legume cover crops are also used in order to sustain or enhance soil health through fixing nitrogen in the atmosphere, encouraging microbial activity, decreasing soil erosion, inhibiting weeds—practices particularly effective in organic and conservation agriculture (Hoyt and Hargrove 1986).

Legumes play an important economic role in Thailand that goes beyond the immediate nutritional value of the product. They are a source of livelihood to small farmers and even indigenous people like the Karen people who use different legumes as food, medicine and materials. The multifunctional nature of legumes offers a boost to the rural economies and culture preservation (Sutjaritjai et al. 2019). Legumes are susceptible to a number of illnesses brought on by nematodes, bacteria, fungus, oomycetes, and viruses. These infections cause severe economic losses by lowering agricultural yields and degrading seed quality (Pande 2009; Rubiales et al. 2015). Table 2 illustrates the disease‐causing viruses, their carriers, signs and symptoms of diseases in legumes and source of transmission.

**TABLE 2:** Disease‐causing viruses in legume‐based plants, their carriers, signs and symptoms of diseases in legumes and source of transmission.

Viruses	Legume carriers	Sign & symptoms	Transmitted via	References
Yellow mosaic virus in mung beans	Common beans, soybean, cowpeas, mung beans, black gram, and pigeon peas	Curling leafs stunted and yellow mosaic patterns	Whiteflies	Mishra et al. (2020)
Yellow mosaic Indian virus in mung beans	Common beans, soybean, peanuts, cowpeas, mung beans, black gram, lablab beans, lima beans, and pigeon peas	Reduced leaf size, stunted growth, shriveled and malformed seeds, and bright yellow mosaic patterns on the leaves	Whiteflies	Kumar et al. (2017)
The virus that causes tomato leaf curl	Common beans and soybeans	Reduced leaf size, upward leaf curling, stunting, and interveinal chlorosis	Whiteflies	Chakraborty (2008)
Yellow leaf curl virus in tomatoes	Common beans, and cowpeas	Reduced fruit production, limited growth, yellowing and curling leaves and a bushy look	Whiteflies	Lapidot and Polston (2006)
Cucumber mosaic virus	Common beans, soybean, peanuts, cowpeas, mung beans, and lima beans	Chlorosis, necrosis, mottling, stunting, distorted leaves, and mosaic patterns	Aphids and seed transmission	Abdullahi et al. (2001)
The peanut stunt virus	Soybean and peanut	Vein clearing, leaf rolling, mosaic pattern, chlorosis, and stunting	Aphids	Hill and Whitham (2014)
Blotchy mosaic virus in soybeans	Soybean	Stunting, smaller leaves, moderate mottling, deformed pods, shortened petioles, crinkled leaves, and chlorotic patches	Aphids	Lamprecht et al. (2010)
Sterility mosaic virus 1 in pigeon peas	Pigeon pea	Stunting, bushy growth, smaller leaves, leaf mosaic, excessive vegetative growth, and ring spots	The eriophyid mite	Patil and Kumar (2015)
Sterility mosaic virus 2 in pigeon peas	Pigeon pea	Stunting, sterility, interveinal chlorosis, and mosaic patterns on the leaves	The eriophyid mite	Patil and Kumar (2015)
Yellow mottle mosaic virus in soybeans	Soybean, mung beans, black gram	Mottling of the leaves, a patchwork of light and dark green patches, stunting and fewer pods	Transmission through seeds	Sandra et al. (2020)
The tobacco streak virus	Soybean, peanuts, mung beans, black gram	Necrotic rings and streaks, distorted leaves, stunting, wilting, and bud blight	Thrips and transfer through seeds	Reddy et al. (2002)
The milk vetch dwarf virus	Cowpeas, mung beans, and lablab beans	Leaf rolling, crinkling, yellowing, stunting, mosaic and dwarfism	Aphids	Zhang et al. (2017)
Ringspot virus in groundnuts	Peanut, soybean	Yellowing, stem necrosis, bronzing, mosaic and mosaic with ringspots	Thrips	Camelo‐García et al. (2014)
Vein necrosis virus in soybeans	Soybean	Yellowing in the vicinity of leaf veins before reddish‐brown lesions develop	Transmission through seeds	Groves et al. (2016)
Common mosaic virus in beans	Common beans, soybean, peanuts, cowpeas, mung beans, black gram, lablab beans, and Bambara groundnuts	Green vein banding, leaf deformation and curling, mosaic patterns, and stunted growth	Pollen, seeds and aphids	Worrall et al. (2015)
Mosaic virus in soybeans	Soybean	Raised puffs and puckering; necrosis of the petioles and stems, bud necrosis, vein clearing in the upper trifoliate leaves, downward curling of the leaf margins	Aphids non‐specific transmission and seed transmission	Rehman et al. (2021)
Yellow common mosaic virus in soybeans	Soybean	Stunting, distortion, and mottling of the leaves	Aphids	Wei et al. (2021)

Nonetheless, market forces like grain selling price, harvesting effort, and cost of pest control may affect the economic feasibility of the use of legumes in some of the farming systems (Yap et al. 2016). Thailand is a country where legumes have to contend with significant biotic stress factors, including fungal infections such as fungal wilt and rusts, which can result in devastating losses in yield. The management of the disease requires the combination of approaches such as the use of resistant legume crops, use of biological control agents, crop rotation, and rational fertilization. Novel approaches to genetic resources use, breeding, and biotechnology, such as genomic selection and gene editing techniques such as CRISPR/Cas9, are being developed to improve the resistance of legumes to pathogens and pests, which offer long‐term solutions to sustainable production of legumes (Aguilar et al. 2025; Bhowmik et al. 2021; Osuna‐Caballero et al. 2024; Sampaio et al. 2020).

Legumes play important ecosystem services of agroecosystems of Thais. Legumes increase the availability of nitrogen in the soil, increase nutrient cycling and promote the diversity of soil microbes through symbiotic relationships with nitrogen‐fixing rhizobia and arbuscular mycorrhizal fungi. This symbiosis increases development and growth resistance to abiotic stresses in crops. Another aspect is the use of root exudates of legume: flavonoids are also used in signaling and interactions between microbes and plants, which lead to benefits in nodulation and intercropping. This environmental service promotes diversified agricultural systems, which do not depend on agrochemicals and agrobiodiversity (Aguilar et al. 2025; Leoni et al. 2021; Lupwayi et al. 2006; Swarnalakshmi et al. 2020).

The problem of climate change is a major threat to the production of legumes in Thailand because the abiotic stressors are heat, drought, salinity and altered rainfall distribution that have endangered the quality and yields of legumes. Legumes are highly susceptible to fluctuations especially at reproductive stages where they suffer greatly due to high temperature which can significantly lower the seed output. Mitigating these effects can be done through breeding of stress resistant strains, application of climate resistant agricultural methods and exploiting useful soil microbes such as arbuscular mycorrhizal, which increase the tolerance of hosts to environmental stresses. The current research efforts are aimed at discovering stress‐adaptive genes, metabolic pathways and creating cultivars that would be resistant to even more developed climate conditions using genomic and biotechnological methods (Araújo et al. 2014; Loo et al. 2022; Mousavi‐Derazmahalleh et al. 2018; Ozga et al. 2016). In a nutshell, Thai legume production is part of the sustainable production, which provides a nutritional, economic, and ecological base. Solutions to issues like disease pressure and climate change, which will include the use of integrated pest management, genetic improvement and improved knowledge about legume‐microbe relationships will be essential in ensuring sustainability. With further promotion of ecological practices and developments in biotechnology and breeding, the Thai legume will help to meet the needs of multiple roles of food security, environmental sustainability, and rural livelihoods (Aguilar et al. 2025; Gogoi et al. 2018; Liu et al. 2020; Mishra et al. 2018; Rajput et al. 2024; Yap et al. 2016).

## 4 Phytochemical Composition of Thai Legumes

Legumes have a high concentration of phenolic compounds, such as isoflavones, biochanin A, daidzein, formononetin, and genistein. Such isoflavones are known to be present in different species of legumes and have been linked with anti‐oxidant, anti‐inflammatory and hormone modulating effects. As an example, studies that have investigated temperate perennial legumes (e.g., Medicago spp., clovers) have demonstrated that younger plants have a higher concentration of isoflavones than those that are fully flowering and that leaves tend to have the highest levels of phytochemicals compared to other plant parts. The Thai legumes, most commonly used in traditional medicine and in food (including *Glycine max* and Vigna spp.) are likely to contain similar compounds (Butkute et al. 2018). Some other bioactive phytochemicals that are found in legumes are tannins, saponins, flavonoids, and polysaccharides. These confer diverse functional‐health effects such as anti‐cancer and anti‐diabetic effects, and cardiovascular protection. Indicatively, chickpeas ( *Cicer arietinum* ), a legume that have received extensive research on its nutritional and functional characteristics, are sources of protein, fiber, vitamins, minerals and other phytochemicals including anti‐nutritional factors such as phytates and lectins. Most of the processing technologies used in Thailand, including soaking and fermentation have the potential to decrease these antinutrients and increase the bioavailability of health‐promoting compounds (Bai et al. 2023; Kumar et al. 2025). It should be emphasized that this inference is extrapolated largely from temperate perennial legumes, and that direct phytochemical quantification of Thai‐grown specimens remains limited. Available Thailand‐based analyses, for example of fermented soybean products and Thai Curcuma‐associated preparations, confirm appreciable phenolic and isoflavone content; however, comprehensive, species‐specific profiling of indigenous Thai varieties under local agro‐climatic conditions is still required to substantiate these claims with primary experimental data.

Climate and elevation are ecological factors that can impact phytochemical diversity, which implies that Thai eco‐climate conditions can impact the chemical profile of native and cultivated legume species. As an example, eco‐climate predictive studies of phytochemical diversity indicate the certain groups of chemicals and active compounds change depending on the elevation and the habitat, and this may be relevant to legumes that originate and cultivated in different parts of Thailand (Tiwari et al. 2023). Another interesting role of rhizobial inoculation in phytochemical enrichment of legumes is also mentioned; legume antioxidants, phenolics, flavonoids, fatty acids and many more have been reported to be increased inoculation with rhizobial. Since the role of symbiotic nitrogen fixation is essential when growing the legumes in Thailand, the bioaugmentation can also enhance the functional traits of the Thai legumes (Silva et al. 2017). Moreover, even leguminous proteins, for example, Glycinin and B‐conglycinin of soybean, can be considered as nutritional proteins, but they also have a role in binding and transporting bioactive substances and stabilizing them and improving their absorption. The giver property implies the possibility of the further nutraceutical usage of Thai legume proteins in food and medicine (Yang et al. 2021).

Overall, it can be predicted that Thai legumes will have an intricate phytochemical profile with a high level of isoflavones, phenolics, flavonoids, and saponins as well as other bioactive substances to support their nutritional and medicinal values. Species features, part of the plant, growth stage, environmental factors, and agronomic procedures like rhizobial inoculation and processing procedures would likely determine their phytochemical profiles. Increased knowledge and profiling of such compounds in the legumes of Thailand may further justify and increase their application in nutrition, traditional medicine, and functional food sectors.

## 5 Medicinal Properties of Thai Legumes

Thai legumes and other vegetarian foods contain abundant various bioactive compounds that confer considerable health effects, such as antioxidant, anti‐inflammatory, anti‐diabetic, anti‐microbial, cardioprotective, anti‐cancer, and neuroprotective effects. Figure 1 shows the ethnomedicinal properties of Thai legumes.

**FIGURE 1:**
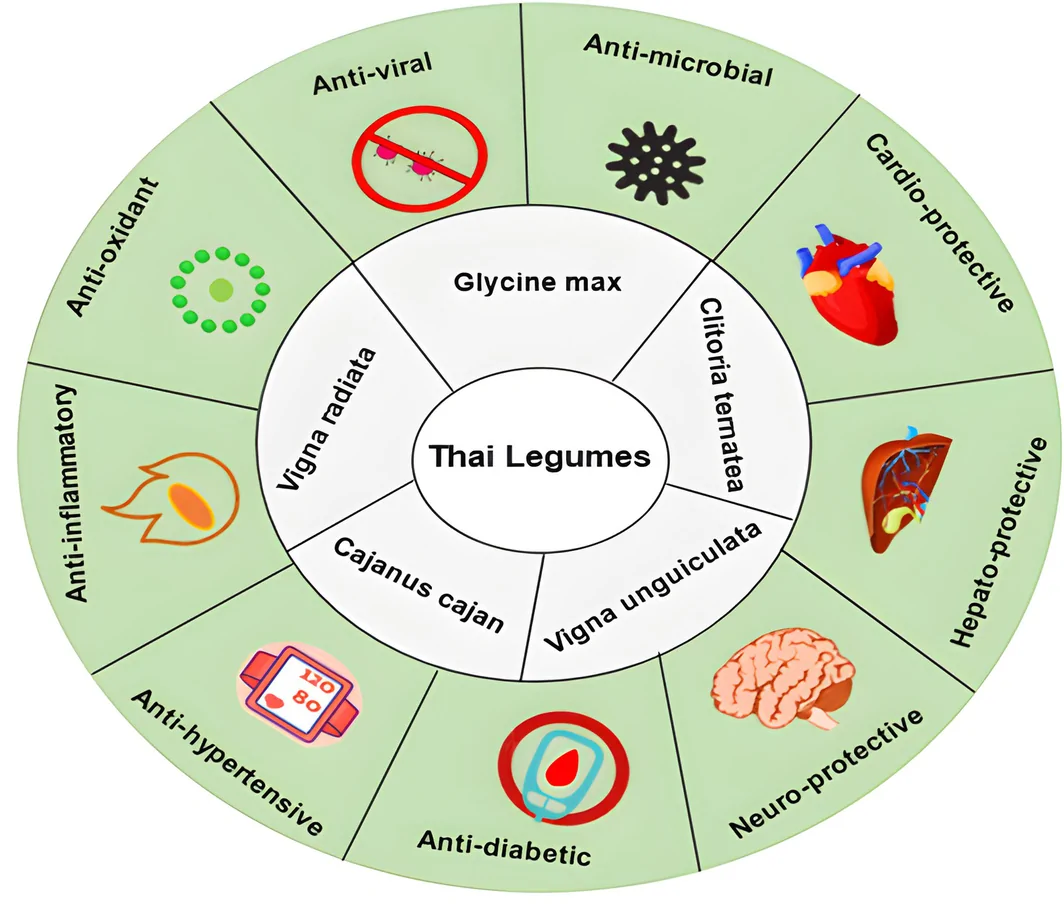
Ethnomedicinal properties of Thai legumes.

### 5.1 Antioxidant Activity

Prominent features of phytochemicals in legumes including phenolics, flavonoids, isoflavones, and bioactive peptides feature antioxidant activity. Such compounds neutralize free radicals, decreasing oxidative stress that contributes to chronic diseases. As an example, investigations of Thai Curcuma species and fermented legumes demonstrate that they contain high amounts of phenolic compounds and radical scavenging properties, which form the basis of their possible usage as a functional food with antioxidant effects. Rhizobial bacteria inoculation of legumes also contributes to their antioxidant capacity and boosts the desirable phytochemicals such as phenolics and flavonoids (Burapan et al. 2020; Deveci et al. 2023; Silva et al. 2017).

### 5.2 Anti‐Inflammatory Activity

Legumes bioactives have been proven to have anti‐inflammatory effects. Inflammatory pathways associated with isoflavones and flavonoids present in legumes suppress pro‐inflammatory mediators and cytokines (Hassan et al. 2023). Legumes bioactives have been proven to have anti‐inflammatory effects (Hou et al. 2024). Randomized controlled clinical trial evidence of plant‐based bioactive substances, such as genistein and curcumin, has shown that they are effective in the treatment of chronic inflammation. The therapeutic utility of legumes can also be mentioned because of their traditional use as antiphlogmatives in the context of folk medicine (Deveci et al. 2023; Josa et al. 2024; Maroyi 2023).

### 5.3 Antidiabetic

The antidiabetic effects of legumes take place in several ways. The gut microbiota may also represent an important pathway linking plant‐derived bioactive compounds with metabolic health, as demonstrated by studies showing modulation of intestinal microbiota and barrier function in the context of type 2 diabetes (Su et al. 2023). A‐amylase and a‐glucosidase inhibitors are components which block the digestion of carbohydrates to reduce the glucose after a meal. Angiotensin‐converting enzyme‐inhibitory bioactive peptides help in enhancing insulin sensitivity. The clinical evidence suggests that intake of legumes or bioactive substances in legumes lowers the glycemic response and helps treat diabetes Type 2. Moreover, these antidiabetic effects can be additionally improved by fermentation and natural processing (Deveci et al. 2023; Josa et al. 2024; Martín‐Cabrejas 2018; Li et al. 2022).

### 5.4 Antimicrobial Activity

Another important characteristic is antimicrobial activity, where legume phytochemicals have been shown to be effective in bacteria, fungi, and viruses. Isoflavones, saponins, and polyphenols interfere with the growth and development of microbes into biofilms. Fermented legume foods use healthy microbes like Lactobacillus and Bacillus species, which add additional antimicrobial effects, which contributes to the health of the gut and food security (Deveci et al. 2023).

### 5.5 Cardio Protective Activity

The cardioprotective actions are a result of the lipid‐lowering, antioxidant, and anti‐inflammatory bioactive of legumes. Flavonoids and phenolic compounds are involved in the regulation of blood pressure, decrease lipid peroxidation, and enhancement of endothelial activity. Extract with high amounts of curcumin, genistein, and allicin have been shown to have better cardiovascular markers, and vasodilation. All of these effects minimize the risks of atherosclerosis and hypertension (Josa et al. 2024; Zou et al. 2021).

### 5.6 Anticancer Activity

Legumes have antioxidant phytochemicals and bioactive peptides that have anticancer properties due to their ability to control the cell cycle, induce apoptosis, and prevent tumor growth. Genistein is one of the isoflavones with chemoprotective effects. Concentration of these anti‐carcinogenic compounds is increased by processes of germination and fermentation. The traditional medicine of legumes and other plants in their nature of adjuvants in cancer prevention and treatments is justified (Aguilar et al. 2025; Gogoi et al. 2018; Liu et al. 2020; Mishra et al. 2018; Rajput et al. 2024; Yap et al. 2016).

### 5.7 Neuroprotective Effects

Legume bioactive has neuroprotective properties that includes antioxidant and anti‐inflammatory action to protect neuronal cells against oxidative stress and neuro‐inflammation that cause neurodegenerative diseases. Although there are few studies on the topic of Thai legumes, the ubiquitous presence of the isoflavones and phenolic with proven neuroprotective properties indicated potential opportunities of future research and development of functional foods (Martirosyan and Miller 2018). To conclude the bioactive properties of Thai legumes, have a wide range of health‐promoting properties. They have anti‐oxidant, anti‐inflammatory, anti‐diabetic, anti‐microbial, cardio‐protective, anti‐cancer and neuro‐protective properties and they are used in functional foods and traditional medicines. These positive effects can be further intensified by improved bioavailability via fermentation, rhizobial inoculation, and improved delivery system, that is, phytosomes, resulting in application integration in nutrition and therapeutics. Further studies are justified as it would help clarify the detailed bioactive patterns of Thai legumes species and confirm their health‐promoting capabilities (Burapan et al. 2020; Deveci et al. 2023; Josa et al. 2024; Silva et al. 2017).

## 6 Industrial Applications of Thai Legumes

Thai legumes have great industrial potential in various developmental food systems, particularly in the functional food industry, plant‐based proteins, and the development of fermented products, due to the nutritional value and flexibility to the new processing technologies. Figure 2 shows the industrial applications of Thai legumes.

**FIGURE 2:**
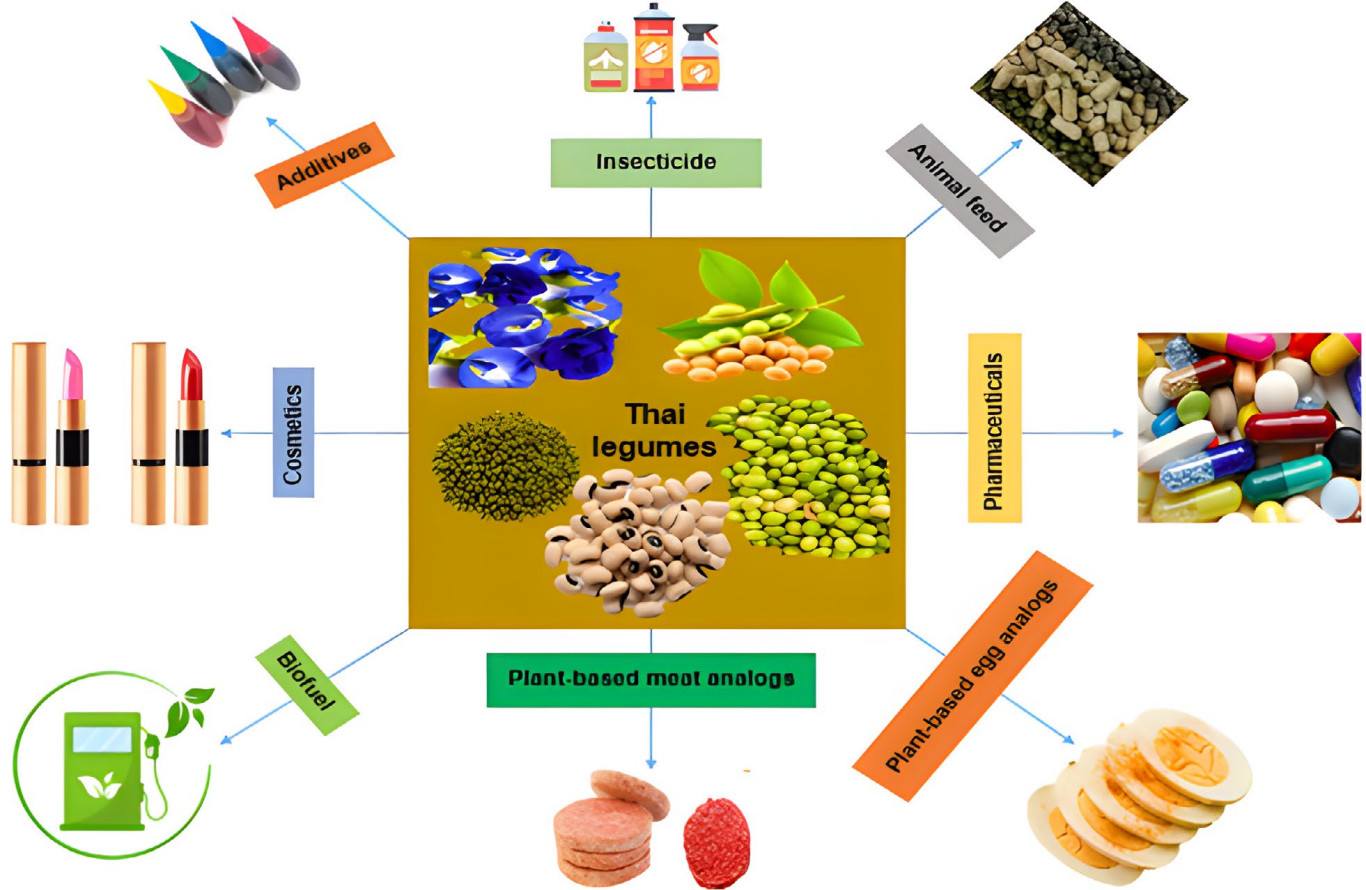
Industrial applications of Thai legumes.

### 6.1 In Food Industry

#### 6.1.1 Functional Foods

Extrusion technology has played a central role in the processing of functional foods. Thai and other kinds of legumes are subjected to extrusion cooking to create ready‐to‐cook and ready‐to‐eat food items like flour, snack foods, gluten‐free products, and meat analogs. Extrusion increases digestibility, reduces or removes anti‐nutritional activities, and increases the functional characteristics of legume proteins and fibers, which has made these products desirable to health‐conscious consumers and increased the consumption of legumes in developing and developed nations. As well, extrusion is considered to be a sustainable and environmentally friendly process since it is cost‐effective, efficient in the course of the process, and produces minimal effluent, which is important in modern conditions and the need to achieve sustainable food production (Faliarizao et al. 2024; Fu et al. 2026).

Another worthwhile method that can be used to enhance the nutritional value and functionality of legumes is solid‐state fermentation (SSF). SSF using fungi like Pleurotus Ostreatus has proved to raise the protein levels of lentils, chickpeas, and soybeans while lowering anti‐nutritional levels like phytate and tannins. SSF also increases anti‐oxidant capacity and protein digestibility, thereby increasing the bioavailability of vital nutrients. Such enhancements enable the SSF‐processed legumes to be integrated successfully in functional food formulations aimed at enhancing the health of human beings (Noori et al. 2025). Further efforts in the development of functional food include the microbial fermentation of the lactic acid bacteria, Bacillus spp., yeasts, and molds to produce bioactive peptides which are anti‐oxidant, antihypertensive, anti‐diabetic, and immunomodulatory. Managed fermentation with specific starter cultures helps in the making of these peptides in legumes to increase their health value and contribute to their applications as nutraceuticals and functional foods. This potential is being refined by developments in the field of the identification of peptides, their debittering, and optimization of their bioavailability (Chourasia et al. 2022; Dhiman et al. 2025).

#### 6.1.2 Plant‐Based Proteins

Thai legumes (peas, beans, chickpeas, and lentils) are high‐quality and sustainable sources of protein which are becoming more popular as consumers grow disposed to plant‐based diets as a result of environmental and health‐related factors. These legumes have nutritional value and promise to decrease dependence on animal proteins. Innovation in processing, which includes germination, fermentation, and enzyme treatment, yields protein derived in legumes more digestible, less allergic, and processing more functional properties, thus making legume proteins applicable in a variety of food products. By highlighting underutilized and native legumes, it will be possible to enhance food security in the region and promote the diversification of agriculture in Thailand and other related settings (Tiruneh et al. 2025).

#### 6.1.3 Fermented Products

Traditional and modern fermentation of Thai legumes in the development of fermented products produces food with a high bioactive compound, probiotics, and improve nutrient composition. Similar products such as fermented foods with alkaline conditions, which are common in Asia, and use Bacillus spp. to aid digestibility, reduce anti‐nutrients, generate vitamins and have probiotic and post biotic benefit. There is also increased synthesis and bioavailability of polyphenols and peptides which are increased by fermentation and added anti‐oxidant, anti‐inflammatory, and cardio‐protective properties. The products also dietary staples, yet they can be consumed as functional and health‐promoting foods with an emerging market popularity (Liu et al. 2022; Owusu‐Kwarteng et al. 2022; Sharma et al. 2022). The opportunity does not mean that challenges of optimizing industrial processing through maintaining product safety, sensorial quality, consumer acceptance, and scalability of fermentation process are eliminated. The future of fermented foods made of legumes is promising with the introduction of new microbiological biotechnologies including genome editing, strain engineering, and precision fermentation (Didinger and Thompson 2021; Singh et al. 2007).

To conclude, the legumes in Thai versatile in terms of industrial use. They are converted to functional foods and plant‐based protein ingredients using extrusion and fermentation technologies, as well as improving digestibility, nutritional quality, and health‐promoting bioactive compounds. Their functionality and sustainability make them well positioned to satisfy the growing global needs for healthy, eco‐friendly, and functional foods. Further development of the processing techniques and biotechnology of microbes will be crucial to unlocking the commercial and health possibilities of Thai food made from legumes.

### 6.2 In Pharmaceutical Industry

#### 6.2.1 Nutraceutical and Drug Development

Legumes, such as those grown in Thailand are abundant in various phytochemical, including isoflavones, phenolics, flavonoids and bioactive peptides which have anti‐oxidant, anti‐inflammatory, anti‐microbial, anti‐diabetic, cardio‐protective and anti‐cancer actions. Such multifunctional bioactivities give the basis to the design of nutraceutical preparations to prevent disease and to promote health in particular chronic diseases like cardiovascular illness, diabetes, cancer, and inflammatory diseases. Bioactive compounds produced by legumes can be used as functional foods and nutraceutical supplements to complement conventional treatments because they have the ability to regulate metabolic processes, oxidative stress, and immune response (Liu et al. 2022; Owusu‐Kwarteng et al. 2022; Sharma et al. 2022). The pharmaceutical potential of legume bioactives could be exploited with the development of drug development procedure, in which the active compounds are isolated and characterized, and the molecular pharmacology of the compounds understood with respect to the mode of action such a strategy is the same as other effective explorations of bioactive natural products in botanical and fungi, where phytochemicals act as lead molecules or inspirations of synthetic analogs with improved efficacy and safety profiles. The discovery of bioactive isoflavones and peptides with distinct enzyme inhibitory properties or receptor modulatory effects opens the potential of new therapeutic agents of Thai legumes (Hannan et al. 2021; Yang et al. 2021).

The formulation sciences can be enhanced through technological methods that can be enhance the stability, bioavailability, and targeted delivery of legume bioactives through the use of encapsulation technologies based on lyposomes or nano‐carriers. Regulated release, protection of sensitive compounds against degradation, and improved absorption are essential issues in the development of functional food and nutraceutical products by the release systems. These innovations make sure that the health values of compounds of legumes are optimally realized and constantly proved to food matrices or supplements (Ajeeshkumar et al. 2021; Kailasapathy 2009). To further improve the nutraceutical value of legumes, fermentation technologies such as solid‐state fermentation and microbial fermentation are used to increase the concentration of bioactive compounds in legumes, minimize anti‐nutrients and in some cases produces novel peptides that are beneficial to health. Fermented legume foods have the potential to be functional foods containing probiotics and bioactive metabolites; therefore, they combine therapeutic potential and nutritional benefits. Applications of useful microorganisms have also been investigated on their potential to synthesize anti‐microbial peptides and metabolic enzymes, which have the potential to be used in food safety, intestinal health, and potential drug discovery (Chourasia et al. 2022; Noori et al. 2025; Raphel and Halami 2024). The disparity between the in vitro and preclinical results and clinical practice is still high. Toxicity, pharmacokinetics, herb‐drug, and efficacy studies during extensive research on Thai legume boactives are necessary to move translationally towards approving nutraceuticals or pharmaceutical development of bioactives. The regulatory framework are becoming more aware of the potential of nutraceuticals as a supplement to conventional drug therapies but demand a high level of evidence to guarantee their safety and consistent effects (Hannan et al. 2021; Sharma et al. 2024). A critical appraisal of this translational gap is warranted. The disparity between promising in vitro or preclinical findings and confirmed clinical benefit arises from several converging factors: much of the bioactivity data derives from crude extracts rather than standardized, chemically characterized fractions; doses effective in cell or animal models frequently exceed concentrations physiologically attainable in humans; the oral bioavailability of isoflavones and phenolic peptides is limited by poor solubility and extensive first‐pass metabolism; and inter‐individual variation in gut microbiota strongly influences the conversion of precursors such as daidzein into active metabolites. Few Thai legume bioactives have progressed beyond exploratory studies, owing to limited funding, the absence of standardized botanical reference materials, and regulatory requirements for reproducible, dose‐defined evidence. To date, most registered human trials involving legume isoflavones concern soy‐derived preparations for menopausal, cardiometabolic and bone‐health endpoints rather than indigenous Thai varieties, and adequately controlled clinical trials on Thai legume preparations are largely lacking. Bridging this gap will require pharmacokinetic and pharmacodynamic characterization, standardized extract preparation, and adequately powered randomized controlled trials conducted on Thai‐grown material.

To sum up, Thai legumes have great potential in drug development and nutraceuticals use as a result of harnessing their extensive phytochemical diversity using the latest extraction, bioprocessing, and formulation technologies. They can be used as preservation of diseases and possible therapeutic agents. Incorporation of phyto‐chemistry, pharmacology, food science, and clinical validation in the future through interdisciplinary studies will be important to ensure the maximum possible use of Thai legume bioactive compounds are fully utilized and commercialized (Ajeeshkumar et al. 2021; Chourasia et al. 2022; Hannan et al. 2021; Kailasapathy 2009; Martín‐Cabrejas 2018; Noori et al. 2025).

### 6.3 In Cosmetic Industry

Thai legumes and other plant‐derived foods are also increasingly considered as bioactive substances in cosmetic products due to their natural antioxidants and anti‐aging properties, and this renders them useful ingredients in nutria‐cosmetic and cosmeceutical products. These phenolics, flavonoids, proteins, and fibers are potent antioxidants that can be used to regulate molecular processes that cause skin aging, suppress oxidative stress, and suppress inflammation, thus resulting in healthier younger skin (Deveci et al. 2023; Hernandez et al. 2020). Legumes and other natural antioxidants are natural sources of reactive oxygen species (ROS) scavengers that lead to skin aging by collagen damage and inflammation. These compounds prevent oxidative damage of skin cells and aid collagen synthesis, thereby preserving skin elasticity and hydration and may help decrease wrinkles. There are few definite study reports on the effect of Thai legume‐derived cosmetics; however, the extensive availability of bioactive phytochemicals in legumes is a good indication of their effective use in anti‐aging effects (Antonopoulou et al. 2016; Hoang et al. 2021).

Cosmetic ingredients that contain other natural ingredients with well‐documented anti‐aging and antioxidant effects are useful in providing parallels. As an illustration, spirulina is a source of phycobiliproteins and carotenoids, which stimulate collagen synthesis and inhibit the breakdown of elastin; grape and winery by‐products contain plentiful phenolic compounds, which stimulate hydration and UV protection in skin; coffee silverskin is rich in chlorogenic acids and caffeine: it inhibits skin‐aging enzymes; red palm oil contains carotenoids and tocopherols which support the barrier function of the skin, and mushrooms are rich in polyps. Cosmetic industries are experiencing difficulties to maintain the stability and bioavailability of these natural antioxidants in the formulations and this is being overcome by encapsulation technologies, enzymatic modification, and nanotechnologies that improve delivery and efficacy in skin application. This is essential to get optimum protective and revitalizing properties of bioactives of legume in anti‐aging products (Antonopoulou et al. 2016; Kailasapathy 2009).

In short, Thai legumes are a promising natural source of antioxidants which can be used as the development of effective anti‐aging cosmetic ingredients. The introduction of the bioactive to the skin care formulation assisted by the latest extraction and delivery technology will help to provide a reflection of the natural, sustainable and health enhancing cosmetic products that help to reduce oxidative skin damage, collagen preservation, and even the visible aging effects. To make the most of these advantages, it is recommended that more research should be conducted to isolate and characterize individual compounds of leguminescent origin to be used in cosmetics (Antonopoulou et al. 2016; Hoang et al. 2021; Hernandez et al. 2020).

### 6.4 Biomaterials and Packaging

Thai legumes are an important and renewable material to advance in biomaterials, especially bioplastic and edible films that can counter the growing environmental issues of the conventional petroleum‐based plastics (Hossain et al. 2026). These legumes contain high amounts of proteins, polysaccharides, lipids, vitamins, and minerals so that they can serve as raw materials to develop biodegradable and compostable packaging solutions with high ecological benefits. Studies show that bioplastics of legume source, which are mostly protein and polysaccharide fractions, have desirable film‐forming properties and tensile strength but generally suffer because of issues like water sensitivity and barrier. In order to improve performance, strategies such as enzymatic crosslinking (such as the use of transglutaminase), mixing with other biopolymers, and using nanomaterial have been used. These modifications enhance mechanical resistance, solubility, and other factors that enhance preservation of packaged food items (Linares‐Castañeda et al. 2023; Porta et al. 2011).

The bioplastics and edible film processing mechanisms used in processing legume‐based products include both laboratory‐scale methods such as solvent casting and scalable industrial processing such as extrusion and compression molding. Reductions in formulation and processing ensure the optimization of the functional properties that address the particular packaging requirements and guarantee the environmental compliance and cost‐effectiveness. New bioactive compounds such as plant extracts and natural antioxidants can be incorporated, as well to create active or smart packaging that increases the shelf life of food (Faliarizao et al. 2024; Linares‐Castañeda et al. 2023).

Legume proteins and polysaccharides‐derived edible films have other advantages: they provide a protective coating that prevents the transfer of oxygen and moisture and therefore, spoilage and preservation of food of high quality. Their biodegradation and harmless edibility offer a dual role of packaging and nutritional supplementation; furthermore, being gluten free and dietary. The most important factor affecting the physicochemical, mechanical, and barrier properties of such films is processing parameters and bioactive compounds incorporation (Porta et al. 2011; Thamarsha et al. 2024). Environmentally, the Thai legume materials manufactured help in reducing the effects of plastic pollution since they are biodegradable and compostable in favorable circumstances. They depend on renewable agricultural sources in their production and have a less carbon footprint than soil‐based plastics. Additionally, the local legume crops should be valorized as a way to promote the circular economy through the conversion of agro‐residues into value‐added biomaterials, which promotes sustainable rural development (Mu et al. 2025; Rujnić‐Sokele and Pilipović 2017).

To conclude, bioplastics and edible films made of Thai legumes leverage their compositional diversity to develop useful, renewable and earth‐friendly alternatives to packaging. Numerous further innovations in crosslinking enzymes, composite formulations, and processing technologies will break the existing limitations, which will further develop these legume‐based biomaterials as viable alternatives to conventional plastic in food packaging processes (Linares‐Castañeda et al. 2023; Porta et al. 2011; Rujnić‐Sokele and Pilipović 2017; Thamarsha et al. 2024). Table 3 illustrates the legume‐based film packaging and their benefits.

**TABLE 3:** Legume‐based film packaging and their benefits.

Legumes	Additive compounds	Film production procedure	Benefits	References
Soybean protein isolates	Glycerol	Distilled water combined with SPI. The plasticizer was glycerol, and 1 N of sodium hydroxide was employed to correct the pH. Before pouring, the solution was heated to 85°C for 15 min. It was then let to sit for 5 min to allow the bubbles to evaporate	Tensile strength and film thickness increased as the SPI concentration increased. Both elongation at break and Young's modulus were reduced. On the other hand, reduced thickness and tensile strength were the results of increased plasticizer concentration	Nandane and Jain (2018)
Soybean protein isolates	Rapeseed oil	A 10% (w/w) concentration of SPI powder was dissolved in distilled water while being agitated at 250 rpm. 1 M sodium hydroxide was used to brimg the pH down. After 20 min of heating at 70°C, then the solution was cooled at 23°C	Reduction in tensile characteristics and WVP	Galus (2018)
Guar gum	Litchishell extracts (LSE), halloysite nanotubes (HNTs), and carboxymethyl cellulose (CMC)	In 100 mL of water, 0.25 g of HNT were distributed. After adding guar gum (0.50 g) and CMC (0.25 g), the mixture was agitated for 4 h at 27°C. After adding glycerol (0.75 g) as a plasticizer, LSE (1%–20% v/v) was added. The mixture was cast onto petri plates sonicated for 20 min, stirred at 50°C and dried for 16 h at 50°C	Enhanced mechanical characteristics, including opacity, elongation at break, film thickness, and UV barrier property	Deshmukh et al. (2022)
Soybean protein isolates	Sorbitol	10% (w/w) PPI solution were added to make the films, and plasticizers including 3%–7% glycerol and 4%–8% sorbitol. The mixture was vacuum‐degassed after the pH was changed to 7.0, 9.0 or 11.0 for casting, 11 g of solution were placed into polystyrene petri dishes and allowed to dry for 12 h at room temperature	Tensile strength, elastic modulus, piercing strength, and elongation at break were all noticeably higher in sorbitol‐plasticized films	Kowalcyzk (2014)
Legume flour	Glycerol	Aqueous solution containing 55 w/v lentil flour agitated at 500 rpm for 10 min. After adding pure glycerol to obtain final concentrations of 1%, 1.5%, and 2% w/v, the mixture was further stirred for 10 min. Using 0.1 M of NaOH the pH was brought to 10.7 after 30 min of heating at either 60°C or 90°C, the solutions were ultrasonically degassed for 10 min at 40 kHz. After pouring ten milliliters of the solution onto LDPE petri dishes, the films were dried at 40°C for 60 h until they were readily peeled off	Lentil flour films had mechanical qualities similar to those of other biodegradable films, were very transparent, and had low water solubility and WVPs. Higher glycerol concentrations resulted in hydrophilic, more flexible films. Compared to films heated at 70°C, those heated at 90°C were stiffer, more transparent and yellower	Aydogdu et al. (2018)
Protein of mung beans	Glycerol, sorbitol and polyethylene glycol‐400	Film solutions were made by dissolving freeze‐dried mung bean proteins in distilled water (3 h/100 mL) the PH was brought down to 9.5. Next, plasticizers (polyethylene glycol‐400, sorbitol and glycerol) were introduced at protein percentage of 30%,40%, 50% and 60%. The components were heated to 75°C for 30 min and homogenized at 10,000 rpm for 2 min. After cooling to room temperature and vacuuming out any remaining air, the mixture was transferred to nonstick pans to set. Films were separated off when trays were cooled to room temperature after being stored at 55°C for the entire night	Tensile strength decreased while elongation at break and WVP rise with increasing plasticizer content	Wittaya (2012)
Protein of mung beans	Glycerol	3.5%–5.0% w/w of mung bean starch was dissolved in distilled water. As a plasticizer, glycerol was used. The mixture was stirred at 750 rpm and heated at 85°C for 30 min to gelatinize the starch. After cooling to 50°C it was sonicated for 20 min to eliminate bubbles. After pouring 40 mL of the casting solution onto an acrylic plate, it was dried for 20 h at 35°C. before testing the films were peeled and kept in a desiccator for 48 h at 28°C and 48.23% RH	Tensile strength, elastic modulus, and oxygen permeability decreased as plasticizer content increased, although elongation, solubility, WVP, and seal strength increased	Rompothi et al. (2017)
Protein of mung beans	Glycerol citric acid	One gram of mung bean protein, 3 g of glycerol and 20 mL of distilled water were combined and heated at 85°C for 1 h while being continuously stirred. After adding varying amount of citric acid (0–150 mg) the mixture was heated to 100°C for 5 min. The solution was put into plastic petri dishes and allowed to dry for 16 h at 30°C after cooling to 50°C	Tensile strength and seal strength increased but WVP and oxygen permeability decreased when compared to mung bean protein films	Yao et al. (2022)

### 6.5 Agricultural Applications

Thai legumes are also important to sustainable agriculture, since they have been reported to fix nitrogen biologically (BNF) or through symbiotic bacteria; rhizobia, which develop root nodules on legumes and can fix atmospheric nitrogen into plant‐accessible forms. This relationship improves the soil nitrogen contents, cutting short the reliance on synthetic nitrogen manure, which is both expensive and harmful to the environment. The inclusion of legumes in the cropping systems therefore enhances the soil fertility, improves the cycling of nutrients, and creates healthier ecosystems of the microbes, thereby resulting in improvement of the soil texture and water retention. Such advantages create strong agroecosystems in terms of increasing biodiversity, supporting useful insects, and natural pest control agents, not to mention providing sustainable farming practices (Kalyan et al. 2024; Kebede 2021; Kokkini et al. 2025). The efficacy of the BNF depends on the legume species, weather conditions, and farm management methods, and fodder legumes have higher rates of nitrogen fixation compared to grain legumes. It is necessary to ensure that the nitrogen input of legumes because of legume genotype is maximized, and appropriate legume genotypes which are resistant to abiotic stresses such as droughts, salinity, and extreme temperatures common in areas such as Thailand are selected. Moreover, the inoculation with efficient rhizobia strains and planting in crop rotations, intercropping, alley, and green manure systems are also important measures that maximize the fixation of nitrogen. These practices can not only increase the yields of next or related non‐legume crops but also decrease the amount of wasted nitrogen and the pollution of the environment (Ghafoor et al. 2024; Kebede 2021). In addition to fixing nitrogen, the legumes are also important to sustainable agriculture because of their capacity to improve the overall health of the soil, improve soil microbial diversity, and improve soil structure. Their presence reduces the cyclic effect of pests and diseases in the natural system, and in most cases, they reduce the requirement of application of chemical pesticides. Legume‐based intercropping and diversified cropping can improve the ecosystem services like carbon sequestration and minimize greenhouse gas emissions, which can help in countering climate change. These environmental advantages are also accompanied by the enhancement of agricultural output and food security, particularly facing the circumstances of climate variability, enhancing the resilience of the food system (Gogoi et al. 2018; Kokkini et al. 2025; Maitra et al. 2023).

Sustainable agricultural research on legumes focuses on holistic strategies that imply the selection of plant genotype, the introduction of microbial inoculants, and the use of local agronomic management strategies. The progress made consists of breeding drought‐adapted varieties and maximizing management to ensure stable and efficient fixation of nitrogen even in stressful environments. Legumes therefore form part of environmentally sustainable future‐oriented agriculture, especially in semi‐arid tropical areas where sustainable nitrogen status and soil protection are highly demanded (Giller and Cadisch 1995; Islam et al. 2021). The Thai legumes play an important role in sustaining farming and raising biodiversity through enhancing soil fertility, minimizing the volume of synthetic fertilizers, sustaining biodiversity, and boosting farm yield, as well as enhancing climate resilience. Their multifunctional characteristics render them important elements in sustainable agriculture systems in pursuing the dual objectives of environmental protection and food security. Further breeding, microbial biotechnology, and integration of farming systems should be invested in order to reap maximum benefits (Ghafoor et al. 2024; Gogoi et al. 2018; Kalyan et al. 2024; Kebede 2021; Kokkini et al. 2025).

## 7 Conclusion

Particularly in the Thai context, legumes of the Fabaceae family are a multipurpose group of plants with significance in traditional medicine, industrial innovation and nutrition. This review emphasizes the abundance of bioactive substances like phenolics, flavonoids, isoflavones and peptides found in Thai legumes, such as *Glycine max* , *Vigna radiata* , *Cajanus cajan* , *Clitoria ternatea* and *Vigna unguiculata* . These substances support their long‐standing use in ethnomedicine and their growing role in functional health applications by contributing to a variety of pharmacological activities, such as anti‐oxidant, anti‐inflammatory, anti‐diabetic, anti‐microbial, cardio‐protective, anti‐cancer and neuroprotective effects. Thai legumes have considerable industrial potential in addition to their medicinal value. The use of food processing technologies, such as fermentation and extrusion, in functional foods, plant‐based protein products, and nutraceutical formations has increased. Their contribution to pharmaceutical development is growing, especially when it comes to the discovery and distribution of bioactive substances with potential for therapeutic use. Additionally, their use in biodegradable biomaterials and sustainable products. Their role in soil health and biological nitrogen fixation in agriculture highlights their significance in climate‐resilient and sustainable farming system. To fully realize their potential, a number of issues need to be resolved. Standardization of phytochemical profiling, thorough toxicological and clinical research, enhanced processing scalability, and increased integration of traditional knowledge with contemporary scientific methods are some of these. Future studies should focus on cutting‐edge extraction techniques, methods for improving bioavailability, and interdisciplinary cooperation between pharmacology, biotechnology and food science. All things considered, Thai legumes have great potential as sustainable resources for industrial growth, environmental sustainability, and health promotion, which calls for further research and development.

## 8 Way Forward

Realizing the full medicinal and industrial potential of Thai legumes will require a coordinated, multidisciplinary research agenda. Priority should be given to: (i) systematic, species‐specific phytochemical profiling of Thai‐grown germplasm under local agro‐climatic conditions, to replace inferences currently drawn from temperate species; (ii) expanded, quantitative ethnobotanical surveys across Thailand's regions and ethnic groups, applying indices such as use‐value and informant consensus factor to prioritize species for validation; (iii) pharmacokinetic and pharmacodynamic characterization followed by adequately powered, dose‐defined randomized controlled trials on standardized Thai legume preparations, to close the translational gap between preclinical and clinical evidence; (iv) development of scalable, standardized processing (e.g., controlled solid‐state fermentation and extrusion) and stable delivery systems to improve bioavailability; and (v) integration of breeding, microbial biotechnology and conservation of indigenous germplasm to secure both agronomic resilience and pharmaceutical value. Strengthening collaboration among ethnobotanists, food scientists, pharmacologists and industry, supported by dedicated funding and standardized reference materials, will be essential to translate traditional knowledge into validated, commercially viable products. Future studies should also incorporate robust statistical and regression‐based modeling approaches to optimize extraction and processing parameters, thereby improving reproducibility, scalability, and the recovery of bioactive compounds (Tao et al. 2026).

## Author Contributions

**Muhammad Tayyab Arshad:** writing – original draft, writing – review and editing, visualization, validation, methodology. **Farhang Hameed Awlqadr:** writing – original draft, writing – review and editing, investigation, conceptualization, visualization. **Iffat Ullah:** writing – review and editing, writing – original draft, resources, supervision, formal analysis. **Mamoona Shehzadi:** writing – original draft, writing – review and editing, validation, formal analysis, supervision. **Aneeq Ahmad:** writing – review and editing, writing – original draft, visualization, validation, conceptualization. **Ali Ikram:** writing – review and editing, writing – original draft, conceptualization, project administration, supervision. **Maneeba Khalid:** writing – original draft, writing – review and editing, visualization, validation, methodology. **Md. Sakhawot Hossain:** writing – original draft, writing – review and editing, investigation, conceptualization, resources.

## Funding

This research was funded by the Graduate School, Prince of Songkla University, through a PSU‐Graduate Studies Scholarship in the year 2025 under contract no. PSU_GSS 2568‐023. Instrumental and laboratory support was provided by the Functional Food and Nutrition Program, Faculty of Agro‐Industry, Prince of Songkla University, Hatyai, Songkhla, 90110, Thailand.

## Conflicts of Interest

The authors declare no conflicts of interest.

## Data Availability

The data that support the findings of this study are available from the corresponding author upon reasonable request.

## References

[fsn372343-bib-0001] Abdullahi, I. , T. Ikotun , S. Winter , G. Thottappilly , and G. I. Atiri . 2001. “Investigation on Seed Transmission of Cucumber Mosaic Virus in Cowpea.” African Crop Science Journal 9, no. 4: 677–684. 10.4314/acsj.v9i4.27589.

[fsn372343-bib-0002] Aguilar, C. H. , D. Pires , C. Cortaga , et al. 2025. “Harnessing Legume Productivity in Tropical Farming Systems by Addressing Challenges Posed by Legume Diseases.” Nitrogen 6, no. 3: 65. 10.3390/nitrogen6030065.

[fsn372343-bib-0003] Ajeeshkumar, K. K. , P. A. Aneesh , N. Raju , M. Suseela , C. N. Ravishankar , and S. Benjakul . 2021. “Advancements in Liposome Technology: Preparation Techniques and Applications in Food, Functional Foods, and Bioactive Delivery: A Review.” Comprehensive Reviews in Food Science and Food Safety 20, no. 2: 1280–1306. 10.1111/1541-4337.12725. 33665991

[fsn372343-bib-0004] Antonopoulou, I. , S. Varriale , E. Topakas , U. Rova , P. Christakopoulos , and V. Faraco . 2016. “Enzymatic Synthesis of Bioactive Compounds With High Potential for Cosmeceutical Application.” Applied Microbiology and Biotechnology 100, no. 43: 6519–6543. 10.1007/s00253-016-7647-9. PMC4939304 27276911

[fsn372343-bib-0005] Araújo, S. S. , S. Beebe , M. Crespi , et al. 2014. “Abiotic Stress Responses in Legumes: Strategies Used to Cope With Environmental Challenges.” Critical Reviews in Plant Sciences 34, no. 1–3: 237–280. 10.1080/07352689.2014.898450.

[fsn372343-bib-0006] Aydogdu, A. , E. Kirtil , G. Sumnu , M. H. Oztop , and Y. Aydogdu . 2018. “Utilization of Lentil Flour as a Biopolymer Source for the Development of Edible Films.” Journal of Applied Polymer Science 135, no. 23: 46356. 10.1002/app.46356.

[fsn372343-bib-0007] Bai, Q. , M. Li , J. Zhou , et al. 2023. “Influence of Processing Methods on Phytochemical Composition of Different Varieties of Beans ( *Phaseolus vulgaris* ).” Food Reviews International 40, no. 7: 1941–1979. 10.1080/87559129.2023.2245026.

[fsn372343-bib-0009] Bhowmik, P. , D. Konkin , P. Polowick , et al. 2021. “CRISPR/Cas9 Gene Editing in Legume Crops: Opportunities and Challenges.” Legume Science 3, no. 3: e96. 10.1002/leg3.96.

[fsn372343-bib-0010] Bruneau, A. , L. P. De Queiroz , J. J. Ringelberg , et al. 2024. “Advances in Legume Systematics 14. Classification of Caesalpinioideae. Part 2: Higher‐Level Classification.” PhytoKeys 240: 1–552. 10.3897/phytokeys.240.101716. PMC11188994 38912426

[fsn372343-bib-0011] Burapan, S. , M. Kim , Y. Paisooksantivatana , B. E. Eser , and J. Han . 2020. “Thai Curcuma Species: Antioxidant and Bioactive Compounds.” Food 9, no. 9: 1219. 10.3390/foods9091219. PMC7555267 32887356

[fsn372343-bib-0012] Butkute, B. , A. Padarauskas , J. Ceseviciene , L. Taujenis , and E. Norkeviciene . 2018. “Phytochemical Composition of Temperate Perennial Legumes.” Crop and Pasture Science 69, no. 10: 1020–1030. 10.1071/cp18206.

[fsn372343-bib-0013] Camelo‐García, V. M. , E. F. B. Lima , P. J. Mansilla‐Córdova , J. A. M. Rezende , E. W. Kitajima , and M. Barreto . 2014. “Occurrence of Groundnut Ringspot Virus on Brazilian Peanut Crops.” Journal of General Plant Pathology 80, no. 3: 282–286.

[fsn372343-bib-0014] Chakraborty, S. 2008. “Tomato Leaf Curl Viruses From India.” In Encyclopedia of Virology. Elsevier.

[fsn372343-bib-0015] Chourasia, R. , L. Chiring Phukon , M. M. Abedin , S. Padhi , S. P. Singh , and A. K. Rai . 2022. “Bioactive Peptides in Fermented Foods and Their Application: A Critical Review.” Systems Microbiology and Biomanufacturing 3, no. 1: 88–109. 10.1007/s43393-022-00125-4.

[fsn372343-bib-0016] Deshmukh, R. K. , K. Akhila , D. Ramakanth , and K. K. Gaikwad . 2022. “Guar Gum/Carboxymethyl Cellulose Based Antioxidant Film Incorporated With Halloysite Nanotubes and Litchi Shell Waste Extract for Active Packaging.” International Journal of Biological Macromolecules 201: 1–13. 10.1016/j.ijbiomac.2021.12.198. 34998867

[fsn372343-bib-0017] Deveci, G. , E. Çelik , D. Ağagündüz , E. Bartkiene , J. M. F. Rocha , and F. Özogul . 2023. “Certain Fermented Foods and Their Possible Health Effects With a Focus on Bioactive Compounds and Microorganisms.” Fermentation 9, no. 11: 923. 10.3390/fermentation9110923.

[fsn372343-bib-0018] Dhiman, S. , S. Kaur , B. Thakur , P. Singh , and M. Tripathi . 2025. “Nutritional Enhancement of Plant‐Based Fermented Foods: Microbial Innovations for a Sustainable Future.” Fermentation 11, no. 6: 346. 10.3390/fermentation11060346.

[fsn372343-bib-0019] Didinger, C. , and H. J. Thompson . 2021. “Defining Nutritional and Functional Niches of Legumes: A Call for Clarity to Distinguish a Future Role for Pulses in the Dietary Guidelines for Americans.” Nutrients 13, no. 4: 1100. 10.3390/nu13041100. PMC8066616 33801714

[fsn372343-bib-0020] Faliarizao, N. , J. D. J. Berrios , and K. D. Dolan . 2024. “Value‐Added Processing of Food Legumes Using Extrusion Technology: A Review.” Legume Science 6, no. 2: e231. 10.1002/leg3.231.

[fsn372343-bib-0116] Fu, J. , X. Fu , P. Wang , Y. Fu , and C. Chen . 2026. “Ultrasonic Degradation of Dendrobium Officinale Polysaccharide: Kinetics, Structural Modification, and its Impact on Wheat Starch Digestibility.” Food Research International 231: 118776. 10.1016/j.foodres.2026.118776 41819928

[fsn372343-bib-0021] Galus, S. 2018. “Functional Properties of Soy Protein Isolate Edible Films as Affected by Rapeseed Oil Concentration.” Food Hydrocolloids 85: 233–241. 10.1016/j.foodhyd.2018.07.026.

[fsn372343-bib-0022] Ghafoor, A. Z. , H. H. Javed , H. Karim , et al. 2024. “Biological Nitrogen Fixation for Sustainable Agriculture Development Under Climate Change–New Insights From a Meta‐Analysis.” Journal of Agronomy and Crop Science 210, no. 5: e12754. 10.1111/jac.12754.

[fsn372343-bib-0023] Giller, K. E. , and G. Cadisch . 1995. “Future Benefits From Biological Nitrogen Fixation: An Ecological Approach to Agriculture.” Plant and Soil 174: 255–277. 10.1007/978-94-011-0053-3_13.

[fsn372343-bib-0024] Gogoi, N. , K. K. Baruah , and R. S. Meena . 2018. “Grain Legumes: Impact on Soil Health and Agroecosystem.” In Legumes for Soil Health and Sustainable Management, 511–539. Springer Singapore. 10.1007/978-981-13-0253-4_16.

[fsn372343-bib-0025] Grdeń, P. , and A. Jakubczyk . 2023. “Health Benefits of Legume Seeds.” Journal of the Science of Food and Agriculture 103, no. 11: 5213–5220. 10.1002/jsfa.12585. 36988580

[fsn372343-bib-0026] Groves, C. , T. German , R. Dasgupta , D. Mueller , and D. L. Smith . 2016. “Seed Transmission of Soybean Vein Necrosis Virus: The First Tospovirus Implicated in Seed Transmission.” PLoS One 11, no. 1: e0147342. 10.1371/journal.pone.0147342. PMC4718560 26784931

[fsn372343-bib-0027] Gulewicz, P. , C. Martinez‐Villaluenga , M. Kasprowicz‐Potocka , and J. Frias . 2014. “Non‐Nutritive Compounds in Fabaceae Family Seeds and the Improvement of Their Nutritional Quality by Traditional Processing—A Review.” Polish Journal of Food and Nutrition Sciences 64, no. 2: 75–89. 10.2478/v10222-012-0098-9.

[fsn372343-bib-0028] Hainds, M. J. , R. J. Mitchell , B. J. Palik , L. R. Boring , and D. H. Gjerstad . 1999. “Distribution of Native Legumes (Leguminoseae) in Frequently Burned Longleaf Pine (Pinaceae)–Wiregrass (Poaceae) Ecosystems.” American Journal of Botany 86, no. 11: 1606–1614. 10.2307/2656797. 10562251

[fsn372343-bib-0029] Hannan, M. A. , M. A. Rahman , A. A. M. Sohag , et al. 2021. “Black Cumin ( *Nigella sativa* L.): A Comprehensive Review on Phytochemistry, Health Benefits, Molecular Pharmacology, and Safety.” Nutrients 13, no. 6: 1784. 10.3390/nu13061784. PMC8225153 34073784

[fsn372343-bib-0114] Hassan, F. U. , C. Liu , M. Mehboob , et al. 2023. “Potential of Dietary Hemp and Cannabinoids to Modulate Immune Response to Enhance Health and Performance in Animals: Opportunities and Challenges.” Frontiers in Immunology 14: 1285052. 10.3389/fimmu.2023.1285052 PMC10726122 38111585

[fsn372343-bib-0030] Hernandez, D. F. , E. L. Cervantes , D. A. Luna‐Vital , and L. Mojica . 2020. “Food‐Derived Bioactive Compounds With Anti‐Aging Potential for Nutricosmetic and Cosmeceutical Products.” Critical Reviews in Food Science and Nutrition 61, no. 22: 3740–3755. 10.1080/10408398.2020.1805407. 32772550

[fsn372343-bib-0031] Hill, J. H. , and S. A. Whitham . 2014. “Control of Virus Diseases in Soybeans.” In Advances in Virus Research, vol. 90, 355–390. Academic Press. 10.1016/B978-0-12-801246-8.00007-X. 25410106

[fsn372343-bib-0032] Hoang, H. T. , J.‐Y. Moon , and Y.‐C. Lee . 2021. “Natural Antioxidants From Plant Extracts in Skincare Cosmetics: Recent Applications, Challenges and Perspectives.” Cosmetics 8, no. 4: 106. 10.3390/cosmetics8040106.

[fsn372343-bib-0033] Hossain, M. S. , M. A. Wazed , M. S. A. Preya , et al. 2026. “A Comprehensive Review of Biotechnological Innovations in Valorization of Food Waste: Enhancing Nutritional, Techno‐Functional Properties, and Process Optimization for Sustainable Product Development.” Food Frontiers 7, no. 2: e70194. 10.1002/fft2.70194.

[fsn372343-bib-0118] Hou, J. , H. Gong , Z. Gong , et al. 2024. “Structural Characterization and Anti‐Inflammatory Activities of a Purified Polysaccharide From Fruits Remnants of *Alpinia zerumbet* (Pers.) Burtt. et Smith.” International Journal of Biological Macromolecules 267, no. Pt 2: 131534. 10.1016/j.ijbiomac.2024.131534 38636158

[fsn372343-bib-0034] Hoyt, G. D. , and W. L. Hargrove . 1986. “Legume Cover Crops for Improving Crop and Soil Management in the Southern United States.” HortScience 21, no. 3: 397–402. 10.21273/hortsci.21.3.397.

[fsn372343-bib-0035] Islam, M. S. , S. Fahad , A. Hossain , et al. 2021. “Legumes Under Drought Stress: Plant Responses, Adaptive Mechanisms, and Management Strategies in Relation to Nitrogen Fixation.” In Engineering Tolerance in Crop Plants Against Abiotic Stress, 179–207. CRC Press. 10.1201/9781003160717-9.

[fsn372343-bib-0036] Josa, E. , G. Barril , and M. Ruperto . 2024. “Potential Effects of Bioactive Compounds of Plant‐Based Foods and Medicinal Plants in Chronic Kidney Disease and Dialysis: A Systematic Review.” Nutrients 16, no. 24: 4321. 10.3390/nu16244321. PMC11678173 39770942

[fsn372343-bib-0037] Kailasapathy, K. 2009. “Encapsulation Technologies for Functional Foods and Nutraceutical Product Development.” CABI Reviews 2009, no. 33: 1–19. 10.1079/pavsnnr20094033.

[fsn372343-bib-0038] Kalyan, K. , D. Gurjar , B. Kumar , and T. V. Kumar . 2024. “The Role of Grain Legumes in Enhancing Soil Health and Promoting Sustainable Agricultural Practices: A Review.” Journal of Experimental Agriculture International 46, no. 8: 344–354. 10.9734/jeai/2024/v46i82712.

[fsn372343-bib-0039] Kebede, E. 2021. “Contribution, Utilization, and Improvement of Legumes‐Driven Biological Nitrogen Fixation in Agricultural Systems.” Frontiers in Sustainable Food Systems 5: 767998. 10.3389/fsufs.2021.767998.

[fsn372343-bib-0040] Kokkini, M. , I. Gazoulis , M. Danaskos , V. Kontogeorgou , P. Kanatas , and I. Travlos . 2025. “Enhancing Ecosystem Services in Agriculture: The Special Role of Legume Intercropping.” Frontiers in Sustainable Food Systems 9: 1547879. 10.3389/fsufs.2025.1547879.

[fsn372343-bib-0119] Kowalcyzk, L. 2014. MGH to Screen All Patients for Substance Abuse. Boston Globe.

[fsn372343-bib-0041] Kumar, N. , S. Hong , Y. Zhu , et al. 2025. “Comprehensive Review of Chickpea ( *Cicer arietinum* ): Nutritional Significance, Health Benefits, Techno‐Functionalities, and Food Applications.” Comprehensive Reviews in Food Science and Food Safety 24, no. 2: e70152. 10.1111/1541-4337.70152. 40047318

[fsn372343-bib-0042] Kumar, S. , B. Tanti , S. K. Mukherjee , and L. Sahoo . 2017. “Molecular Characterization and Infectivity of Mungbean Yellow Mosaic India Virus Associated With Yellow Mosaic Disease of Cowpea and Mungbean.” Biocatalysis and Agricultural Biotechnology 11: 183–191. 10.1016/j.bcab.2017.07.004.

[fsn372343-bib-0043] Lamprecht, R. L. , G. G. F. Kasdorf , M. Stiller , S. M. Staples , L. H. Nel , and G. Pietersen . 2010. “Soybean Blotchy Mosaic Virus, a New Cytorhabdovirus Found in South Africa.” Plant Disease 94, no. 11: 1348–1354. 10.1094/PDIS-09-09-0598. 30743624

[fsn372343-bib-0044] Lapidot, M. , and J. E. Polston . 2006. “Resistance to Tomato Yellow Leaf Curl Virus in Tomato.” In Natural Resistance Mechanisms of Plants to Viruses, 503–520. Springer Netherlands.

[fsn372343-bib-0045] Leoni, F. , H. Hazrati , I. S. Fomsgaard , A.‐C. Moonen , and P. Kudsk . 2021. “Determination of the Effect of Co‐Cultivation on the Production and Root Exudation of Flavonoids in Four Legume Species Using LC‐MS/MS Analysis.” Journal of Agricultural and Food Chemistry 69, no. 32: 9208–9219. 10.1021/acs.jafc.1c02821. PMC8389803 34346216

[fsn372343-bib-0115] Li, Q. Y. , Z. M. Dou , C. Chen , Y. M. Jiang , B. Yang , and X. Fu . 2022. “Study on the Effect of Molecular Weight on the Gut Microbiota Fermentation Properties of Blackberry Polysaccharides In Vitro.” Journal of Agricultural and Food Chemistry 70, no. 36: 11245–11257. 10.1021/acs.jafc.2c03091 36053142

[fsn372343-bib-0046] Linares‐Castañeda, A. , X. M. Sánchez‐Chino , Y. Gómez , et al. 2023. “Cereal and Legume Protein Edible Films: A Sustainable Alternative to Conventional Food Packaging.” International Journal of Food Properties 26, no. 2: 3197–3213. 10.1080/10942912.2023.2267785.

[fsn372343-bib-0047] Liu, A. , Y.‐S. Ku , C. A. Contador , and H.‐M. Lam . 2020. “The Impacts of Domestication and Agricultural Practices on Legume Nutrient Acquisition Through Symbiosis With Rhizobia and Arbuscular Mycorrhizal Fungi.” Frontiers in Genetics 11, no. 103586: 583954. 10.3389/fgene.2020.583954. PMC7554533 33193716

[fsn372343-bib-0048] Liu, H. , X. Xu , H. Cui , et al. 2022. “Plant‐Based Fermented Beverages and Key Emerging Processing Technologies.” Food Reviews International 39, no. 8: 5844–5863. 10.1080/87559129.2022.2097256.

[fsn372343-bib-0050] Loo, W. T. , K.‐O. Chua , P. Mazumdar , A. Cheng , N. Osman , and J. A. Harikrishna . 2022. “Arbuscular Mycorrhizal Symbiosis: A Strategy for Mitigating the Impacts of Climate Change on Tropical Legume Crops.” Plants (Basel) 11, no. 21: 2875. 10.3390/plants11212875. PMC9657156 36365329

[fsn372343-bib-0051] Lupwayi, N. Z. , G. W. Clayton , and W. A. Rice . 2006. “Rhizobial Inoculants for Legume Crops.” Journal of Crop Improvement 15, no. 2: 289–321. 10.1300/j411v15n02_09.

[fsn372343-bib-0053] Maitra, S. , S. Praharaj , M. Brestic , et al. 2023. “Rhizobium as Biotechnological Tools for Green Solutions: An Environment‐Friendly Approach for Sustainable Crop Production in the Modern Era of Climate Change.” Current Microbiology 80, no. 7: 219. 10.1007/s00284-023-03317-w. 37204538

[fsn372343-bib-0054] Maroyi, A. 2023. “Medicinal Uses of the Fabaceae Family in Zimbabwe: A Review.” Plants 12, no. 6: 1255. 10.3390/plants12061255. PMC10051751 36986943

[fsn372343-bib-0055] Martín‐Cabrejas, M. A. 2018. “CHAPTER 1. Legumes: An Overview.” In Legumes: Nutritional Quality, Processing and Potential Health Benefits, 1–18. Royal Society Of Chemistry. 10.1039/9781788015721-00001.

[fsn372343-bib-0056] Martirosyan, D. , and E. Miller . 2018. “Bioactive Compounds: The Key to Functional Foods.” Bioactive Compounds in Health and Disease 1, no. 3: 36. 10.31989/bchd.v1i3.539.

[fsn372343-bib-0057] Mishra, G. P. , H. K. Dikshit , R. Sv , et al. 2020. “Yellow Mosaic Disease (YMD) of Mungbean ( *Vigna radiata* (L.) Wilczek): Current Status and Management Opportunities.” Frontiers in Plant Science 11: 918. 10.3389/fpls.2020.00918. PMC7327115 32670329

[fsn372343-bib-0058] Mishra, R. K. , A. Bohra , N. Kamaal , et al. 2018. “Utilization of Biopesticides as Sustainable Solutions for Management of Pests in Legume Crops: Achievements and Prospects.” Egyptian Journal of Biological Pest Control 28, no. 1: 3. 10.1186/s41938-017-0004-1.

[fsn372343-bib-0059] Mousavi‐Derazmahalleh, M. , P. E. Bayer , J. K. Hane , et al. 2018. “Adapting Legume Crops to Climate Change Using Genomic Approaches.” Plant, Cell & Environment 42, no. 1: 6–19. 10.1111/pce.13203. PMC6334278 29603775

[fsn372343-bib-0060] Mu, Y. , S. Lv , J. Liu , et al. 2025. “Recent Advances in Research on Biomass‐Based Food Packaging Film Materials.” Comprehensive Reviews in Food Science and Food Safety 24, no. 1: e70093. 10.1111/1541-4337.70093. 39812511

[fsn372343-bib-0061] Nandane, A. S. , and R. K. Jain . 2018. “Optimization of Formulation and Process Parameters for Soy Protein‐Based Edible Film Using Response Surface Methodology.” Journal of Packaging Technology and Research 2, no. 3: 203–210. 10.1007/s41783-018-0045-2.

[fsn372343-bib-0062] Noori, S. M. A. , M. Hojjati , and R. Sorourian . 2025. “Enhancing Nutritional Quality and Functionality of Legumes: Application of Solid‐State Fermentation With Pleurotus Ostreatus.” Food Science & Nutrition 13, no. 11: e70783. 10.1002/fsn3.70783. PMC12552784 41140439

[fsn372343-bib-0063] Osuna‐Caballero, S. , N. Rispail , E. Barilli , and D. Rubiales . 2024. “Management and Breeding for Rust Resistance in Legumes.” Journal of Plant Pathology 108: 35–61. 10.1007/s42161-024-01679-z.

[fsn372343-bib-0064] Owusu‐Kwarteng, J. , D. Agyei , F. Akabanda , R. A. Atuna , and F. K. Amagloh . 2022. “Plant‐Based Alkaline Fermented Foods as Sustainable Sources of Nutrients and Health‐Promoting Bioactive Compounds.” Frontiers in Sustainable Food Systems 6: 885328. 10.3389/fsufs.2022.885328.

[fsn372343-bib-0065] Ozga, J. A. , H. Kaur , R. P. Savada , and D. M. Reinecke . 2016. “Hormonal Regulation of Reproductive Growth Under Normal and Heat‐Stress Conditions in Legume and Other Model Crop Species.” Journal of Experimental Botany 68, no. 8: erw464. 10.1093/jxb/erw464. 28011717

[fsn372343-bib-0066] Pande, S. 2009. “Integrated Foliar Diseases Management of Legumes.” In International Conference on Grain Legumes: Quality Improvement, Value Addition and Trade. https://oar.icrisat.org/.

[fsn372343-bib-0067] Patil, B. L. , and P. L. Kumar . 2015. “Pigeonpea Sterility Mosaic Virus: A Legume‐Infecting Emaravirus From South Asia.” Molecular Plant Pathology 16, no. 8: 775–786. 10.1111/mpp.12238. PMC6638375 25640756

[fsn372343-bib-0068] Porta, R. , L. Mariniello , P. Di Pierro , A. Sorrentino , and C. V. L. Giosafatto . 2011. “Transglutaminase Crosslinked Pectin‐ and Chitosan‐Based Edible Films: A Review.” Critical Reviews in Food Science and Nutrition 51, no. 3: 223–238. 10.1080/10408390903548891. 21390943

[fsn372343-bib-0069] Rajput, A. , Q. Ali Panhwar , and H. Babar . 2024. “Role of Leguminous Crops by Enhancing Soil Fertility and Plant Nutrition.” In Soil Amendments for Sustainability. Intechopen. 10.5772/intechopen.1006827.

[fsn372343-bib-0071] Raphel, S. , and P. M. Halami . 2024. “Bioactive Compounds From Food‐Grade Bacillus.” Journal of the Science of Food and Agriculture 105, no. 8: 4085–4095. 10.1002/jsfa.13935. 39373131

[fsn372343-bib-0072] Reddy, A. S. , R. P. Rao , K. Thirumala‐Devi , et al. 2002. “Occurrence of Tobacco Streak Virus on Peanut ( *Arachis hypogaea* ) in India.” Plant Disease 86, no. 2: 173–178. 10.1094/PDIS.2002.86.2.173. 30823316

[fsn372343-bib-0073] Rehman, F. U. , M. Kalsoom , M. Adnan , et al. 2021. “Soybean Mosaic Disease (SMD): A Review.” Egyptian Journal of Basic and Applied Sciences 8, no. 1: 12–16. 10.1080/2314808X.2021.1881245.

[fsn372343-bib-0074] Rompothi, O. , P. Pradipasena , K. Tananuwong , A. Somwangthanaroj , and T. Janjarasskul . 2017. “Development of Non‐Water Soluble, Ductile Mung Bean Starch Based Edible Film With Oxygen Barrier and Heat Sealability.” Carbohydrate Polymers 157: 748–756. 10.1016/j.carbpol.2016.09.007. 27987987

[fsn372343-bib-0075] Rubiales, D. , S. Fondevilla , W. Chen , et al. 2015. “Achievements and Challenges in Legume Breeding for Pest and Disease Resistance.” Critical Reviews in Plant Sciences 34, no. 1–3: 195–236. 10.1080/07352689.2014.898445.

[fsn372343-bib-0076] Rujnić‐Sokele, M. , and A. Pilipović . 2017. “Challenges and Opportunities of Biodegradable Plastics: A Mini Review.” Waste Management & Research: The Journal for a Sustainable Circular Economy 35, no. 2: 132–140. 10.1177/0734242x16683272. 28064843

[fsn372343-bib-0078] Saensouk, P. , S. Saensouk , S. Mattapha , K. Chanthavongsa , and T. Jitpromma . 2025. “Diversity and Traditional Uses of Fabaceae Species in Maha Sarakham Province, Thailand.” Diversity 17, no. 12: 838. 10.3390/d17120838.

[fsn372343-bib-0079] Sampaio, A. M. , S. D. S. Araújo , D. Rubiales , and M. C. Vaz Patto . 2020. “Fusarium Wilt Management in Legume Crops.” Agronomy 10, no. 8: 1073. 10.3390/agronomy10081073.

[fsn372343-bib-0080] Sandra, N. , A. Tripathi , H. K. Dikshit , B. Mandal , and R. K. Jain . 2020. “Seed Transmission of a Distinct Soybean Yellow Mottle Mosaic Virus Strain Identified From India in Natural and Experimental Hosts.” Virus Research 280: 197903. 10.1016/j.virusres.2020.197903. 32105764

[fsn372343-bib-0081] Seehalak, W. , N. Tomooka , A. Waranyuwat , et al. 2006. “Genetic Diversity of the Vigna Germplasm From Thailand and Neighboring Regions Revealed by AFLP Analysis.” Genetic Resources and Crop Evolution 53, no. 5: 1043–1059. 10.1007/s10722-004-7939-2.

[fsn372343-bib-0082] Sharma, M. S. V. , K. S. Y. Ojha , B. Singh , S. Gupta , and S. K. Pandey . 2024. “The Role of Functional Foods and Nutraceuticals in Disease Prevention and Health Promotion.” European Journal of Nutrition and Food Safety 16, no. 2: 61–83. 10.9734/ejnfs/2024/v16i21388.

[fsn372343-bib-0083] Sharma, R. , B. Diwan , B. P. Singh , and S. Kulshrestha . 2022. “Probiotic Fermentation of Polyphenols: Potential Sources of Novel Functional Foods.” Food Production, Processing and Nutrition 4, no. 1: 21. 10.1186/s43014-022-00101-4.

[fsn372343-bib-0084] Silva, L. R. , C. Bento , A. C. Gonçalves , et al. 2017. “Legume Bioactive Compounds: Influence of Rhizobial Inoculation.” AIMS Microbiology 3, no. 2: 267–278. 10.3934/microbiol.2017.2.267. PMC6605017 31294160

[fsn372343-bib-0086] Singh, R. J. , G. H. Chung , and R. L. Nelson . 2007. “Landmark Research in Legumes.” Genome 50, no. 6: 525–537. 10.1139/g07-037. 17632574

[fsn372343-bib-0113] Su, M. , T. Tang , W. Tang , Y. Long , L. Wang , and M. Liu . 2023. “Astragalus Improves Intestinal Barrier Function and Immunity by Acting on Intestinal Microbiota to Treat T2DM: A Research Review.” Frontiers in Immunology 14: 1243834. 10.3389/fimmu.2023.1243834 PMC10450032 37638043

[fsn372343-bib-0087] Sutjaritjai, N. , P. Panyadee , M. Phumthum , A. Inta , and H. Balslev . 2022. “High Diversity of Medicinal Uses of Thai Legumes (Fabaceae) and Their Potential in Public Herbal Medicine.” Diversity 14, no. 8: 588. 10.3390/d14080588.

[fsn372343-bib-0088] Sutjaritjai, N. , P. Wangpakapattanawong , H. Balslev , and A. Inta . 2019. “Traditional Uses of Leguminosae Among the Karen in Thailand.” Plants 8, no. 12: 600. 10.3390/plants8120600. PMC6963713 31847100

[fsn372343-bib-0089] Swarnalakshmi, K. , V. Yadav , D. Tyagi , D. W. Dhar , A. Kannepalli , and S. Kumar . 2020. “Significance of Plant Growth Promoting Rhizobacteria in Grain Legumes: Growth Promotion and Crop Production.” Plants 9, no. 11: 1596. 10.3390/plants9111596. PMC7698556 33213067

[fsn372343-bib-0117] Tao, Y. , Z. Yu , T. Chen , W. Jin , W. Jin , and L. Yu . 2026. “Large‐Scale Assessment of Regression Modeling Practices in Phytomedicine Extraction Process Optimization.” iScience 29, no. 8: 116883. 10.1016/j.isci.2026.116883 PMC13523843 42666754

[fsn372343-bib-0090] Thamarsha, A. , N. Kumar , P. Pratibha , et al. 2024. “A Review of Functional Properties and Applications of Legume‐Based Edible Coatings.” Legume Science 6, no. 4: 70004.

[fsn372343-bib-0091] Tiruneh, A. , P. Ptaszek , D. Żmudziński , and T. Tarko . 2025. “Peas ( *Pisum sativum* Subsp. Arvense Asch) and Beans ( *Vicia faba* var. Minor) as Source of Quality Plant Proteins.” Molecules 30, no. 9: 2009. 10.3390/molecules30092009. PMC12073127 40363814

[fsn372343-bib-0092] Tiwari, D. , P. Kewlani , K. S. Gaira , I. D. Bhatt , R. C. Sundriyal , and V. Pande . 2023. “Predicting Phytochemical Diversity of Medicinal and Aromatic Plants (MAPs) Across Eco‐Climatic Zones and Elevation in Uttarakhand Using Generalized Additive Model.” Scientific Reports 13, no. 1: 10888. 10.1038/s41598-023-37495-1. PMC10322824 37407604

[fsn372343-bib-0094] Vidhale, D. K. , V. Mora , S. Pasam , A. Kotha , U. Konda , and N. Boggula . 2024. “Critical Review on *Pongamia pinnata* : A Versatile Medicinal Plant.” Asian Journal of Pharmaceutical Research and Development 12, no. 4: 138–146. 10.22270/ajprd.v12i4.1455.

[fsn372343-bib-0095] Wei, Z. Y. , C. Jiang , C. Y. Mao , et al. 2021. “Occurrence of Soybean Yellow Common Mosaic Virus in Soybean in China Showing Yellow Common Mosaic Disease.” Plant Disease 105, no. 4: 1236. 10.1094/PDIS-08-20-1738-PDN.

[fsn372343-bib-0096] Weir, B. S. , S. J. Turner , W. B. Silvester , D.‐C. Park , and J. M. Young . 2004. “Unexpectedly Diverse Mesorhizobium Strains and *Rhizobium leguminosarum* Nodulate Native Legume Genera of New Zealand, While Introduced Legume Weeds Are Nodulated by Bradyrhizobium Species.” Applied and Environmental Microbiology 70, no. 10: 5980–5987. 10.1128/aem.70.10.5980-5987.2004. PMC522066 15466541

[fsn372343-bib-0097] Wittaya, T. 2012. “Protein‐Based Edible Films: Characteristics and Improvement of Properties.” In Structure and Function of Food Engineering. Intech Open.

[fsn372343-bib-0098] Worrall, E. A. , F. O. Wamonje , G. Mukeshimana , J. J. Harvey , J. P. Carr , and N. Mitter . 2015. “Bean Common Mosaic Virus and Bean Common Mosaic Necrosis Virus: Relationships, Biology, and Prospects for Control.” Advances in Virus Research 93: 1–46. 10.1016/bs.aivir.2015.04.002. 26111585

[fsn372343-bib-0099] Yadav, A. K. , R. Kamal , V. Agarwal , and P. K. Sharma . 2025. “Phytosomes: Advancing Herbal Medicine Through Innovative Integration.” Current Pharmaceutical Design 31, no. 42: 3390–3399. 10.2174/0113816128347309250320061045. 40247801

[fsn372343-bib-0100] Yang, R. , L. Zhu , D. Meng , et al. 2021. “Proteins From Leguminous Plants: From Structure, Property to the Function in Encapsulation/Binding and Delivery of Bioactive Compounds.” Critical Reviews in Food Science and Nutrition 62: 5203–5223. 10.1080/10408398.2021.1883545. 33569994

[fsn372343-bib-0101] Yao, S. , B. J. Wang , and Y. M. Weng . 2022. “Preparation and Characterization of Mung Bean Starch Edible Films Using Citric Acid as Cross‐Linking Agent.” Food Packaging and Shelf Life 32: 100845. 10.1016/j.fpsl.2022.100845.

[fsn372343-bib-0102] Yap, V. Y. , A. De Neergaard , and T. B. Bruun . 2016. “‘To Adopt or Not to Adopt?’ Legume Adoption in Maize‐Based Systems of Northern Thailand: Constraints and Potentials.” Land Degradation and Development 28, no. 2: 731–741. 10.1002/ldr.2546.

[fsn372343-bib-0103] Zhang, C. , H. Zheng , D. Yan , et al. 2017. “Complete Genomic Characterization of Milk Vetch Dwarf Virus Isolates From Cowpea and Broad Bean in Anhui Province, China.” Archives of Virology 162, no. 8: 2437–2440. 10.1007/s00705-017-3348-7 28386650

[fsn372343-bib-0104] Zou, L. , D. Wu , G. Ren , et al. 2021. “Bioactive Compounds, Health Benefits, and Industrial Applications of Tartary Buckwheat ( *Fagopyrum tataricum* ).” Critical Reviews in Food Science and Nutrition 63: 657–673. 10.1080/10408398.2021.1952161. 34278850

